# EZH1/2 inhibition augments the anti-tumor effects of sorafenib in hepatocellular carcinoma

**DOI:** 10.1038/s41598-021-00889-0

**Published:** 2021-11-01

**Authors:** Yuko Kusakabe, Tetsuhiro Chiba, Motohiko Oshima, Shuhei Koide, Ola Rizq, Kazumasa Aoyama, Junjie Ao, Tatsuya Kaneko, Hiroaki Kanzaki, Kengo Kanayama, Takahiro Maeda, Tomoko Saito, Ryo Nakagawa, Kazufumi Kobayashi, Soichiro Kiyono, Masato Nakamura, Sadahisa Ogasawara, Eiichiro Suzuki, Shingo Nakamoto, Shin Yasui, Rintaro Mikata, Ryosuke Muroyama, Tatsuo Kanda, Hitoshi Maruyama, Jun Kato, Naoya Mimura, Anqi Ma, Jian Jin, Yoh Zen, Masayuki Otsuka, Atsushi Kaneda, Atsushi Iwama, Naoya Kato

**Affiliations:** 1 Department of Gastroenterology, Graduate School of Medicine, Chiba University, 1-8-1 Inohana, Chuo-ku, Chiba, 260-8670 Japan; 2 Division of Stem Cell and Molecular Medicine, Center for Stem Cell Biology and Regenerative Medicine, The Institute of Medical Science, The University of Tokyo, 4-6-1 Shirokenadai, Minato-ku, Tokyo, 108-8639 Japan; 3 Department of Cellular and Molecular Medicine, Graduate School of Medicine, Chiba University, 1-8-1 Inohana, Chuo-ku, Chiba, 260-8670 Japan; 4 Department of Molecular Virology, Graduate School of Medicine, Chiba University, 1-8-1 Inohana, Chuo-ku, Chiba, 260-8670 Japan; 5 Department of Gastroenterology and Hepatology, Nihon University School of Medicine, 30-1 Oyaguchi-Kamicho, Itabashi-ku, Tokyo, 173-8610 Japan; 6 Department of Gastroenterology, Juntendo University School of Medicine, 2-1-1 Hongo, Bunkyo-ku, Tokyo, 113-08421 Japan; 7 Department of Transfusion Medicine and Cell Therapy, Chiba University, 1-8-1 Inohana, Chuo-ku, Chiba, 260-8670 Japan; 8 Mount Sinai Center for Therapeutics Discovery, Icahn School of Medicine at Mount Sinai, New York, NY 10029 USA; 9 Department of Pharmacological Sciences, Icahn School of Medicine at Mount Sinai, New York, NY 10029 USA; 10 Department of Oncological Sciences, Tisch Cancer Institute, Icahn School of Medicine at Mount Sinai, New York, NY 10029 USA; 11 Institute of Liver Studies, King’s College Hospital, London, UK; 12 Department of General Surgery, Graduate School of Medicine, Chiba University, 1-8-1 Inohana, Chuo-ku, Chiba, 260-8670 Japan; 13 Department of Molecular Oncology, Graduate School of Medicine, Chiba University, 1-8-1 Inohana, Chuo-ku, Chiba, 260-8670 Japan

**Keywords:** Cancer epigenetics, Hepatocellular carcinoma

## Abstract

Both EZH2 and its homolog EZH1 function as histone H3 Lysine 27 (H3K27) methyltransferases and repress the transcription of target genes. Dysregulation of H3K27 trimethylation (H3K27me3) plays an important role in the development and progression of cancers such as hepatocellular carcinoma (HCC). This study investigated the relationship between the expression of EZH1/2 and the level of H3K27me3 in HCC. Additionally, the role of EZH1/2 in cell growth, tumorigenicity, and resistance to sorafenib were also analyzed. Both the lentiviral knockdown and the pharmacological inhibition of EZH1/2 (UNC1999) diminished the level of H3K27me3 and suppressed cell growth in liver cancer cells, compared with EZH1 or EZH2 single knockdown. Although a significant association was observed between EZH2 expression and H3K27me3 levels in HCC samples, overexpression of EZH1 appeared to contribute to enhanced H3K27me3 levels in some EZH2^low^H3K27me3^high^ cases. Akt suppression following sorafenib treatment resulted in an increase of the H3K27me3 levels through a decrease in EZH2 phosphorylation at serine 21. The combined use of sorafenib and UNC1999 exhibited synergistic antitumor effects in vitro and in vivo. Combination treatment canceled the sorafenib-induced enhancement in H3K27me3 levels, indicating that activation of EZH2 function is one of the mechanisms of sorafenib-resistance in HCC. In conclusion, sorafenib plus EZH1/2 inhibitors may comprise a novel therapeutic approach in HCC.

## Introduction

Hepatocellular carcinoma (HCC) is a refractory cancer associated with chronic liver disease caused by persistent viral hepatitis, excessive alcohol abuse, and non-alcoholic fatty liver^1^. HCC is well known as the second most common cancer-related cause of death worldwide^2^. Although diagnostic tools and therapeutic approaches have been devised, the prognosis for patients with advanced HCC remains poor^3^. Therefore, the improvement of these patients is an important and urgent task to be resolved. Systemic chemotherapy using multiple receptor tyrosine kinase inhibitors (MKIs) is an important component for the treatment of advanced HCC. Sorafenib and lenvatinib have been approved and are used as first-line therapy and regorafenib as second-line therapy^4–6^. Although these agents may provide a survival advantage in some cases, the long-term prognosis remains unsatisfactory.

Epigenetics refers to regulatory gene expression mechanisms, which do not involve changes in the DNA sequence. This includes DNA methylation, histone modification, non-coding RNAs, and chromatin remodeling^7,8^. Such mechanisms are tightly involved in various biological phenomena, such as differentiation, development, and tumorigenesis. Importantly, epigenetic machinery dysregulation plays a critical role in the development and progression of various cancers, including HCC^9,10^.

EZH2 is a polycomb group protein (PcG) and a central component of the polycomb repressive complex 2 (PRC2)^11,12^. It catalyzes the trimethylation of histone H3 at lysine 27 (H3K27), thereby repressing the transcription of target genes. Of interest, EZH1 functions as a backup enzyme for EZH2, in the presence of EZH2 dysfunctions in transcriptional repression, both in embryonic and somatic stem cells^13,14^. An increase in EZH2 and H3K27me3 levels has been reported to be associated with aggressive tumor phenotypes and unfavorable prognosis^15,16^. Although these findings indicate that inhibition of EZH1/2 is a potential therapeutic approach for HCC, the significance of the combined use of MKIs and EZH1/2 inhibitors in HCC remains to be elucidated.

In the present study, we performed clinicopathological analyses of surgical HCC samples and loss-of-function assays of EZH1/2 in HCC cells using short hairpin RNA lentiviral vectors and low-molecular-weight compounds. Subsequently, the changes in H3K27me3 levels after sorafenib treatment were investigated. The effects of the combined use of sorafenib and EZH1/2 inhibitors in HCC were analyzed in in vitro and in vivo xenograft transplantation models.

## Results

### Relationship between EZH1/2 expression and H3K27me3 levels in surgical HCC samples

**Figure 1:**
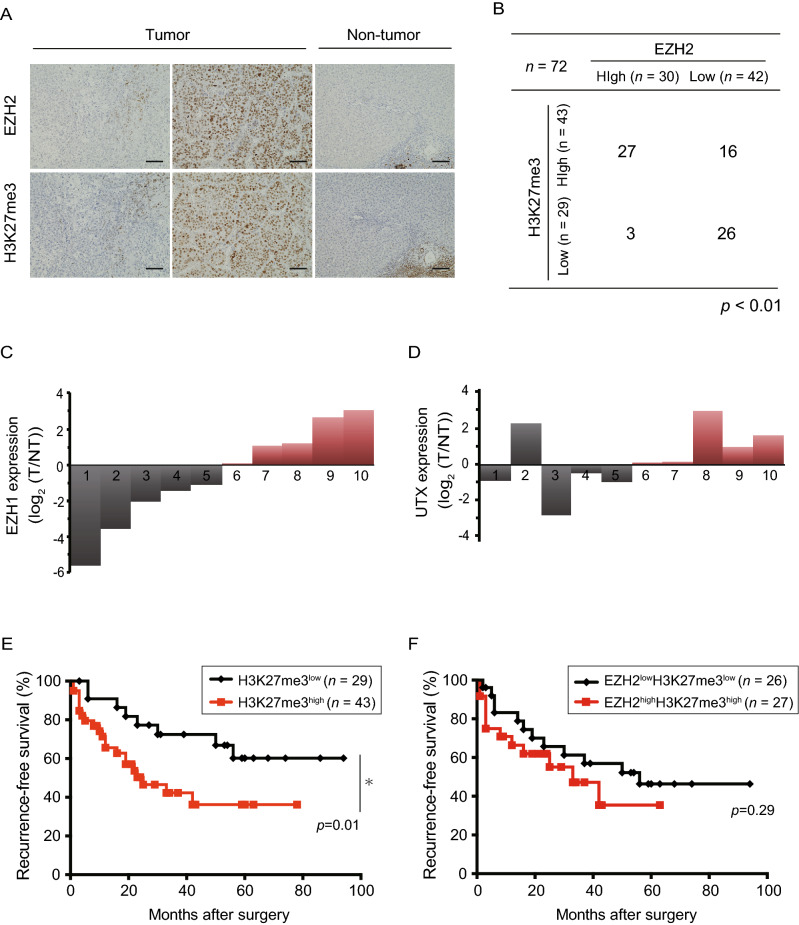
EZH1/2 expression and H3K27me3 levels in surgical HCC specimen. (**A**) Representative images of immunohistochemistry staining for EZH2 and H3K27me3 tumors and tumor-free tissues. Scale bar = 100 µm. (**B**) Relationship between EZH2 expression and H3K27me3 levels in HCC tissues (Fisher’s exact test, *p* < 0.01). (**C**) Waterfall plots showing *EZH1* mRNA expression in EZH2^low^H3K27me3^high^ HCCs. (**D**) Waterfall plots showing *UTX* mRNA expression in EZH2^low^H3K27me3^high^ HCCs. (**E**) Cumulative recurrence-free survival based on H3K27me3 levels (log-rank test, *p < 0.05). (**F**) Cumulative recurrence-free survival between EZH2^low^H3K27me3^low^ and EZH2^high^H3K27me3^high^ patients (log-rank test, *p < 0.05).

To understand the relationship between EZH2 expression and H3K27me3 levels in HCC, immunohistochemical analyses were performed in 72 pairs of primary HCC tissues and corresponding non-tumor tissues. In tumor-free tissues, both EZH2 and H3K27me3 were only detected in infiltrated lymphocytes and periportal hepatocytes. In clear contrast, HCC tissues exhibited varying degrees of increased EZH2 expression and H3K27me3 level (Fig. 1A). Based on the percentage of cells strongly expressing these markers (≥ 50% of tumor cells), tumor tissues were divided into either EZH2^low^ or EZH2^high^ and either H3K27me3^low^ or H3K27me3^high^.

Regarding EZH2 expression, 42 (58.3%) and 30 (41.7%) of 72 samples were classified as EZH2^low^ and EZH2^high^, respectively. Similarly, 29 (40.3%) and 43 (59.7%) samples were classified as H3K27me3^low^ and H3K27me3^high^, respectively. The Fisher’s exact test revealed a significant association between EZH2 expression and H3K27me3 levels (*p* < 0.01) (Fig. 1B). However, 16 samples out of 43 H3K27me3^high^ samples showed low EZH2 expression. Because mRNA from 10 of the 16 EZH2^low^H3K27me3^high^ samples was sufficient and of a high enough quality, they were subjected to RT-qPCR. Four out of 10 samples showed an increase in *EZH1* levels compared to those in non-tumor tissues (Fig. 1C). It is possible that EZH1 rather than EZH2 was associated with the high levels of H3K27me3 in some EZH2^low^H3K27me3^high^ samples. Among the rest samples, 4 of 6 samples showed a decrease in expression levels of *ubiquitously transcribed tetratricopeptide repeat on chromosome X* (*UTX*), a H3K27 demethylase, compared to those in non-tumor tissues (Fig. 1D). These findings suggested that not only *EZH1* overexpression but also *UTX* decreased expression might be attributable to increased H3K27me3 levels in some of the EZH2^low^H3K27me3^high^ cases.

### Evaluation of H3K27me3 levels in clinicopathological features

We then analyzed the differences between the clinicopathological features of H3K27me3^low^ HCCs (n = 29) and H3K27me3 ^high^ HCCs (n = 43) (Table 1). Importantly, H3K27me3^high^ HCCs were significantly related to stage progression (UICC stage III and IV) (*p* = 0.04). They also showed a trend for higher serum levels of a-fetoprotein, which is a diagnostic marker of HCC (*p* = 0.24). We then performed prognostic analyses on these patients, using a Kaplan–Meier survival analysis (Fig. 1E). During the follow-up period (45.6 months), 10 patients died from HCC. The median recurrence-free survival in patients with H3K27me3^low^ HCC and H3K27me3^high^ HCC was “not reached” and 25.0 months, respectively (*p* = 0.02). These results were in agreement with those of previous reports^17^. However, there was no significant difference of PFS between EZH2^low^H3K27me3^low^ and EZH2^high^H3K27me3^high^ patients (Fig. 1F). Collectively, high H3K27me3 levels appeared to be closely associated with HCC prognosis.

**Table 1:** Clinicopathological features of H3K27me3^low^ and H3K27me3^high^ HCC.

Characteristics	H3K27me3 levels		
	Low (*n* = 29)	High (*n* = 43)	*p* value
Age (years)	63.6 ± 12.2	67.9 ± 7.9	0.07
Gender (male/female)	21/8	30/13	0.81
Etiology (HBV/HCV/other)	11/9/9	9/17/17	0.29
Liver cirrhosis (yes/no)	6/23	12/31	0.49
AFP (ng/mL)	1020.7 ± 2372.4	3579.4 ± 11,176.6	0.24
PIVKA-II (mAU/mL)	2966.4 ± 6920.3	8301.4 ± 22,892.9	0.24
Tumor diameter (mm)	43.9 ± 33.6	46.2 ± 33.4	0.78
Portal involvement (yes/no)	5/24	41/29	0.15
Edmondson^b^ grade (I–II/III–IV)	6/23	11/32	0.78
UICC^c^ stage (I–II/III–IV)	22/7	22/21	0.04^a^

^a^Statistically significant.

^b^Edmondson–Steiner.

^c^International Union Against Cancer.

### Basal levels of EZH1/2 and H3K27me3 and loss-of-function assays in liver cancer cells

The basal expression of EZH1/2 and H3K27me3 levels was investigated in four liver cancer cell lines: Huh1, Huh7, HepG2, and PLC/PRF/5. Immunofluorescence staining demonstrated a higher expression and co-localization of EZH2 and H3K27me3 in liver cancer cell nuclei. RT-qPCR revealed that, in these cells, *EZH2* mRNA expression was significantly higher than that of *EZH1* (Supplementary Fig. S1). Among them, Huh7 and HepG2 cells were subjected to the most of the subsequent experiments.

**Figure 2:**
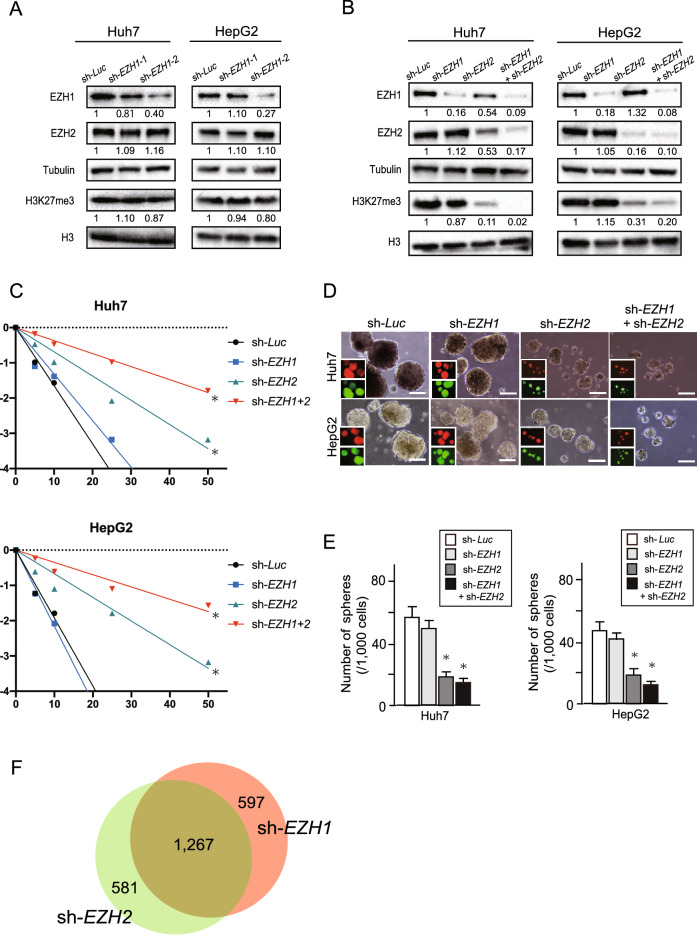
Knockdown of *EZH1* and/or *EZH2* in Huh7 and HepG2 cells. (**A**) Stable *EZH1* knockdown cells were subjected to Western blot analyses. (**B**) Stable *EZH1* and/or *EZH2*-knockdown cells were subjected to Western blot analyses. (**C**) Limiting dilution assay of Huh7 and HepG2 cells transduced with sh-*EZH1*, sh-*EZH2* and sh-*EZH1/2*. The sphere formation frequency was calculated by extreme limiting dilution analysis (*p < 0.05, **p < 0.01). (**D**) Representative bright-field images of spheres formed on day 14 of low attachment cell culture. Fluorescence images are shown in insets. Scale bar = 100 µm. (**E**) Number of large spheres generated from 1000 cells transduced with the indicated viruses on culture day 14 (Student’s t test, *p < 0.05). (**F**) RNA sequence was performed using stable *EZH1* or *EZH2*-knockdown Huh7 cells. Venn diagram depicting the number of genes showing de-repression after *EZH1*- or *EZH2* knockdown.

Next, we conducted loss-of-function assays for EZH1 and EZH2 using lentivirus-mediated shRNA in Huh7 and HepG2 cells, successfully achieving stable knockdown of EZH1 and EZH2 using RFP and EGFP as a viral infection marker, respectively. Two shRNAs were made against *EZH1* (sh-*EZH1-*1 and sh-*EZH1-*2), both markedly repressing EZH1 expression in HCC cells (Fig. 2A). sh-*EZH1-2* was used for subsequent experiments, and sh-*EZH2* was used as previously designed and prepared by our group^18^. While *EZH2-*knockdown remarkably decreased H3K27me3 levels, the decrease caused by *EZH1-*knockdown was minimal (Fig. 2B). In the limiting dilution and sphere formation assay, *EZH2*-knockdown pronouncedly inhibited sphere forming abilities of Huh7 and HepG2 cells (Fig. 2C–E). Importantly, the double knockdown of *EZH1/2* showed an additional inhibitory effect in cell growth and sphere formation in vitro.

Subsequently, the profiling of de-repressed genes (fold change > 2.0) after lentiviral knockdown of *EZH1* or *EZH2* was analyzed in Huh7 cells. The number of genes de-repressed following *EZH1* and *EZH2* knockdown was 1864 and 1848, respectively (Fig. 2F). The number of overlapped genes was 1267, indicating that approximately 30% of target genes of EZH1 and EZH2 was not necessarily identical. Gene set enrichment analysis (GSEA) demonstrated that *EZH1* knockdown cells were significantly enriched for genes involved in mitotic spindle [normalized enrichment score (NES) 1.93, p-value 0, and false discovery rate (FDR) q-value 0], UV response down (NES 1.79, p-value 0, and FDR q-value 0.001), and Hedgehog signaling (NES 1.63, p-value 0, and FDR q-value 0.008). *EZH2* knockdown cells were significantly enriched for genes involved in mitotic spindle (NES 1.83, p-value 0, and FDR q-value 0.002), UV response down (NES 1.77, p-value 0, and FDR q-value 0.003), Hedgehog signaling (NES 1.6, p-value 0.003, and FDR q-value 0.010), K-RAS signaling up (NES 1.74, p-value 0, and FDR q-value 0.003) and interferon alpha response (NES 1.63, p-value 0, and FDR q-value 0.009). Taken together, these results show that simultaneous *EZH1/2* inhibition is required for sufficient inhibition of cell growth ability and tumorigenic activity.

### Pharmacological deletion of EZH1/2 in liver cancer cells

**Figure 3:**
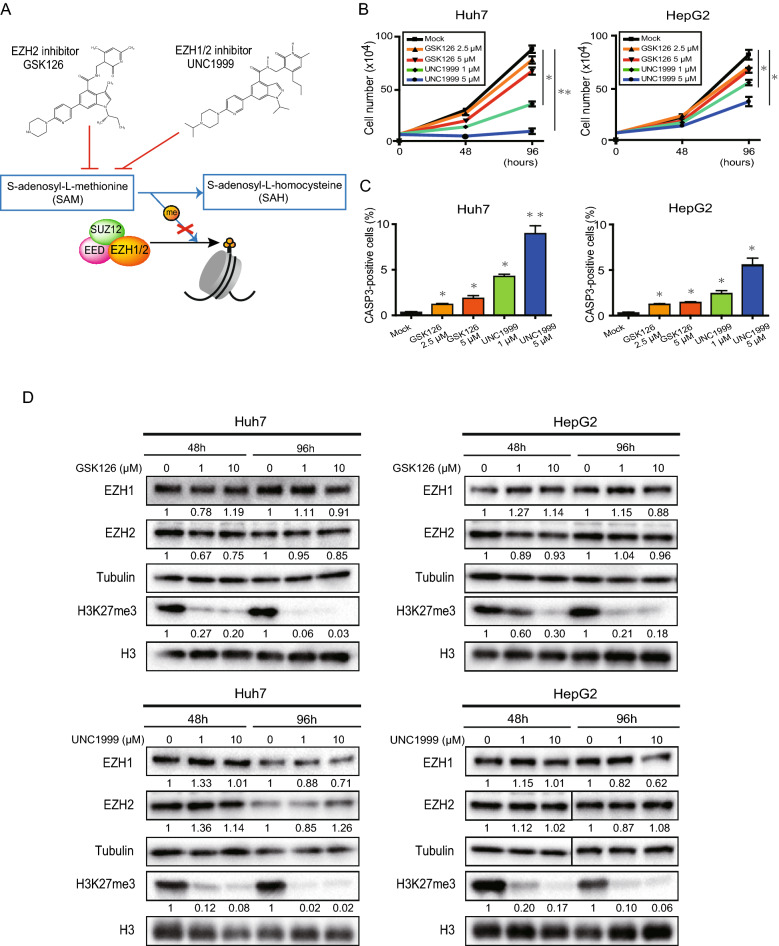
In vitro assays of Huh7 and HepG2 cells treated with GSK126 or UNC1999. (**A**) GSK126 and UNC1999 inhibit S-Adenosyl methionine competitively. (**B**) Cell growth inhibition of HCC cells treated with GSK126 and UNC1999 (repeated measures ANOVA, *p < 0.05, **p < 0.01). (**C**) Quantification of apoptotic cells treated with GSK126 and UNC1999 for 48 h based on percentages of CASP3-positive cells by immunostaining (Student’s t test, *p < 0.05, **p < 0.01). (**D**) Western blot analysis for EZH1, EZH2 and H3K27me3 after treatment of HCC cells with a range of concentrations of GSK126 and UNC1999 for 48 or 96 h. RI was shown as the means of three independent experiments.

Both the EZH2 inhibitor GSK126 and the EZH1/2 dual inhibitor UNC1999 has been developed elsewhere (Fig. 3A)^19,20^. Subsequently, we performed loss-of-function assays of EZH1 and EZH2 using these inhibitors. The cell number in HCC cells treated with UNC1999, but not with GSK126, was significantly lower than those in control cells (Fig. 3B, Supplementary Fig. S2A). Both drugs induced cellular apoptosis in a dose-dependent manner (Fig. 3C, Supplementary Fig. S2B). Of note, UNC1999 exerted a pronounced effect at a low concentration compared with GSK126. Western blotting demonstrated that both GSK126 and UNC1999 apparently reduced H3K27me3 levels in liver cancer cells in a time- and dose-dependent manner (Fig. 3D). Although RIs of H3K27me3 was significantly lower in UNC1999-treated cells than those in GSK126-treated cells for 48 h, there was no significant difference in RIs of H3K27me3 between two groups for 96 h.

### An increase in H3K27me3 levels in liver cancer cells treated with sorafenib

**Figure 4:**
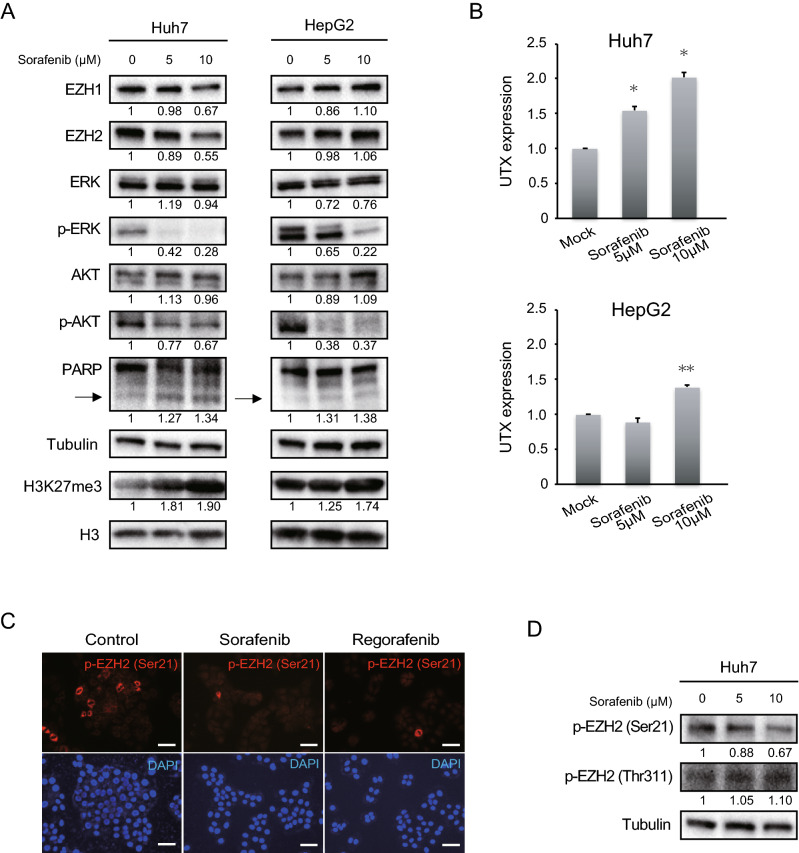
An increase in H3K27me3 levels caused by sorafenib in liver cancer cells. (**A**) Cells treated with sorafenib in varying concentration for 48 h were subject to Western blot analyses. The arrow indicates the cleaved form pf PARP. RI was shown as the means of three independent experiments. (**B**) Real-time PCR analyses of the *UTX* expression in cells treated with sorafenib for 48 h (Student’s t test, *p < 0.01, **p < 0.05) (**C**) Immunocytochemical analyses for phospho-EZH2 at serine 21 (Ser21) (red) and DAPI (blue) after treatment of Huh7 cells with sorafenib (5 μM) and regorafenib (5 μM) for 48 h. Scale bar = 50 µm. (**D**) Cells treated with sorafenib and GSK126 or UNC1999 were subjected to Western blot analyses.

Next, we examined EZH1/2 expression and H3K27me3 levels in liver cancer cells treated with sorafenib. Unexpectedly, Western blotting showed increased H3K27me3 levels and concomitantly decreased phospho-Akt levels (Fig. 4A). However, only minimal changes were observed in the EZH1/2 protein. An increase in levels of cleaved PARP, a marker of apoptosis, was detected in a dose-dependent manner. Since it was possible that sorafenib treatment caused an ubiquitous downregulation of UTX, a demethylase for H3K27, RT-qPCR demonstrated that UTX expression was rather upregulated in a dose-dependent manner (Fig. 4B). It has been reported that Akt phosphorylates EZH2 at serine 21 (Ser21) and inhibits its enzyme activity for H3K27me3^21^. Consistently, immunocytochemical staining analyses using a phospho-specific antibody, revealed that phosphorylation of EZH2 at Ser21 remarkably decreased in HCC cells treated with sorafenib and regorafenib (Fig. 4C), suggesting augmented enzymatic activity of EZH2 upon sorafenib treatment. Concordant with these findings, Western blotting demonstrated that sorafenib treatment resulted in a decrease in phosphorylated levels of EZH2 (Ser21), but not EZH2 at threonine 311 (Thr311) in Huh7 cells in a dose-dependent manner (Fig. 4D). These findings indicate that phosphorylation of EZH2 might occur in a site-specific manner.

### Combination treatment with sorafenib and EZH1/2 inhibitor in liver cancer cells

**Figure 5:**
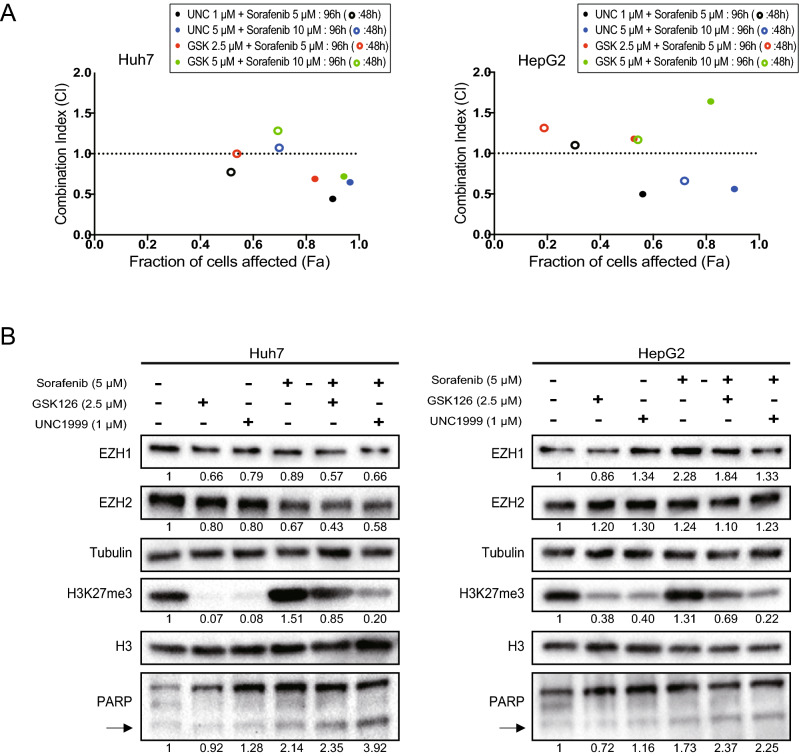
Combined use of sorafenib and GSK126 or UNC1999 in liver cancer cells. (**A**) The combination index was calculated based on the data of the trypan blue dye exclusion test. (**B**) Cells treated with sorafenib and GSK126 or UNC1999 for 48 h were subjected to Western blot analyses. The arrow indicates the cleaved form of PARP. RI was shown as the means of three independent experiments.

Forced expression of *EZH1/2* resulted in an increase in H3K27me3 levels in Huh7 cells (Supplementary Fig. S3A). The inhibitory effect of sorafenib treatment was diminished in *EZH1/2* overexpressed cells compared with control cells (Supplementary Fig. S3B). In clear contrast, proliferation inhibition followed by sorafenib treatment was more effective in *EZH1/2* double knockdown cells than those in control cells (Supplementary Fig. S4). These results implicated that *EZH1/2* knockdown sensitized HCC cells to sorafenib treatment. Therefore, we investigated the effect of simultaneous treatment with sorafenib and EZH2 or EZH1/2 inhibitors on the growth of Huh7 cells. The Combination index (CI) was calculated^22^. The results showed a convincing synergism of the combined use of sorafenib and EZH1/2 inhibitor UNC1999 or EZH2-specific inhibitor GSK126 (CI < 1.0) in Huh7 cells (Fig. 5A). Synergism of the combined use of sorafenib and EZH1/2 inhibitor UNC1999, but not GSK126, was observed in HepG2 cells. EZH2 inhibition by GSK126 and UNC1999 successfully canceled abnormal H3K27me3 changes caused by sorafenib (Fig. 5B). The levels of cleaved PARP were enhanced in combined use of sorafenib and GSK126 or UNC1999.

### Combined use of sorafenib and UNC1999 in vivo xenograft model

**Figure 6:**
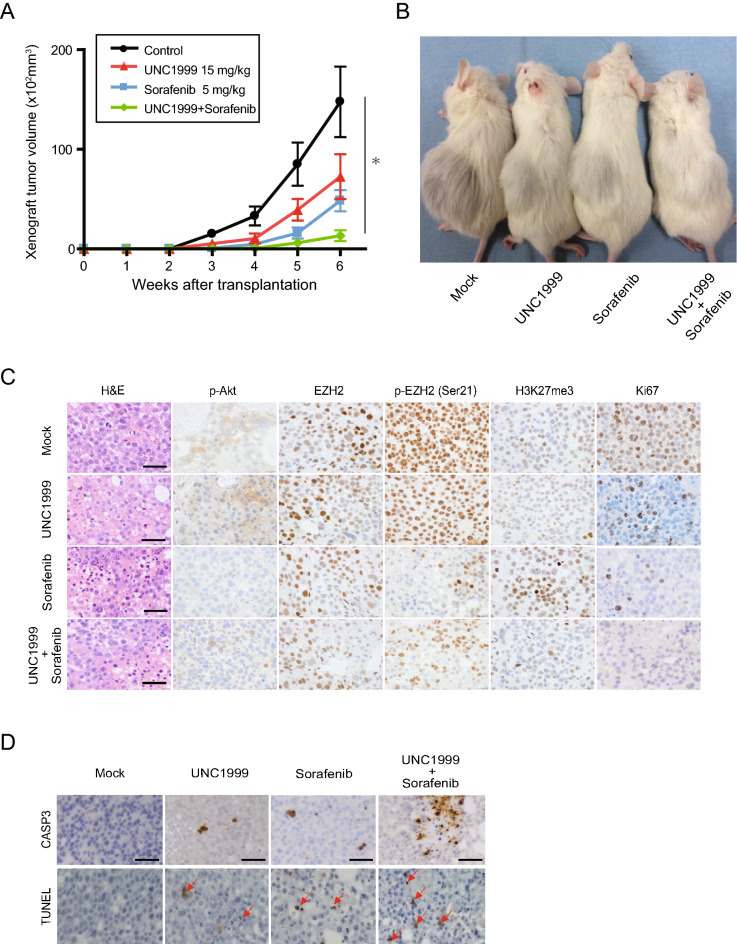
Xenograft transplantation using NOD/SCID mice and histopathological examination of xenograft tumors. (**A**) Tumor growth curve of the recipient mice treated with sorafenib and/or UNC1999 (repeated measures ANOVA, *p < 0.05). (**B**) Representative images of recipient mice treated with sorafenib and/or UNC1999 6 weeks after the transplantation. (**C**) Xenograft tumors were subjected to Hematoxylin and eosin staining and immunohistochemical staining. Scale bar = 50 µm. (**D**) Immunostaining of CASP3 and TUNEL assay. Arrows indicate TUNEL-positive cells. Scale bar = 50 µm.

**Figure 7:**
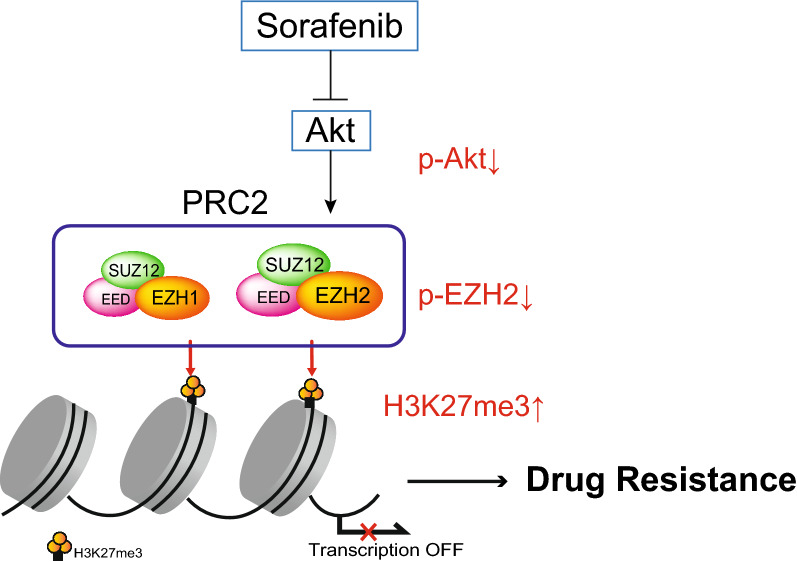
A proposed model for the effect of the combined use of sorafenib and EZH1/2 inhibitors, in terms of H3K27me3 level changes.

We then evaluated the anti-tumor effects of sorafenib and/or UNC1999 in a xenograft transplant model using NOD/SCID mice (Fig. 6A,B). The oral administration of sorafenib and/or UNC1999 (i.e., daily administration of 5 mg/kg of sorafenib and/or 3 days a week administration of 15 mg/kg of UNC1999 for 6 weeks) was started on the days following HCC cells implantation. Subcutaneous tumor growth was significantly suppressed by the concomitant administration of sorafenib and UNC1999, compared to their single administration. Consistent with the results from the in vitro experiments, immunohistochemical analyses of the subcutaneous tumors showed that treatment with sorafenib induced an increase of the H3K27me3 levels, accompanied by a decrease of both the phosphorylated Akt and the phosphorylated EZH2 (Fig. 6C). The combined use of UNC1999 and sorafenib canceled an increase in H3K27me3 levels at least partially. Ki-67 staining revealed that the combined treatment efficiently inhibited cell growth. The Ki-67 labeling index in mock, UNC1999, sorafenib, and combination groups was 47.4%, 11.8%, 7.8%, and 4.7%, respectively. Both CASP3 staining and Terminal deoxynucleotidyl transferase-mediated dUTP nick end-labeling (TUNEL) assay demonstrated that the combination treatment more clearly induced apoptosis compared with mock or single agent treatment (Fig. 6D). Taken together, these results show that the combined use of EZH1/2 dual inhibitor may enhance the anti-tumor effects of sorafenib by canceling sorafenib-induced activation of EZH2 and additional inhibition of EZH1 (Fig. 7).

## Discussion

The PcG proteins are responsible for maintaining the transcriptional repression of target genes through the catalyzation of histone modifications^23^. The PcG gene family members form two major form complexes, PRC1 and PRC2. Both Ezh1 and Ezh2 are the catalytic components of PRC2 and trimethylate H3K27. Although EZH1 partially compensates for EZH2 deficiencies in hematopoietic stem cells, the H3K27me2/3 activity of EZH1 is markedly weaker than that of EZH2^24^.

In in vitro assays, the basal expression of EZH1/2 was exactly detected in the HCC cell lines examined. However, the EZH1 expression was significantly lower than that of EZH2. Although *EZH2* knockdown caused an apparent decrease in H3K27me3 levels, *EZH1* knockdown caused only modest changes in H3K27me3 levels. Consistent with these findings, the inhibition of cell growth and sphere formation was obviously observed in *EZH2* knockdown cells compared to *EZH1* knockdown cells. Importantly, *EZH1/2* double knockdown demonstrated an additional inhibitory effect on cell growth and sphere formation in HCC cells compared to single knockdown. Our clinicopathological analyses of HCC surgical specimens showed a significant association between the expression of EZH2 and H3K27me3 levels, and that the H3K27me3 levels function as a prognostic marker for survival. Interestingly, the aberrant overexpression of *EZH1* and decreased expression of UTX were observed in some HCC cases with high levels of H3K27me3 unexpectedly showing low EZH2 expression (EZH2^low^H3K27me3^high^). Taken together, the results show that not only EZH2 but also EZH1 plays an important role in the development and progression of HCC through the alteration of H3K27me3 levels.

Both GSK126 and UNC1999 inhibit S-Adenosyl methionine (SAM) competitively, thereby inhibiting SAM-dependent methyltransferases, including EZH1/2^25^. Given that the IC50 of EZH1 enzymatic inhibition differs between GSK126 (EZH1; 680 nmol/L and EZH2; 9.9 nmol/L) and UNC1999 (EZH1 45 nmol/L and EZH2 < 10 nmol/L), then UNC1999, but not GSK126, operates as a dual EZH1/2 inhibitor^19,20^. In the present study, UNC1999 inhibited cell growth and apoptosis induction in HCC cells at a low concentration compared with GSK126. Since RNA-seq analyses demonstrated that approximately 30% of the de-repressed genes following EZH1 and EZH2 knockdown are different, dual inhibition of EZH1 and EZH2 might be more potent than EZH2 inhibition alone.

It has been reported that aberrant histone modification is closely involved in drug resistance^26,27^. Enhanced H3K27me3 levels in the gene body of *SLFN11*, a DNA-damage repair gene, contribute to the resistance for etoposide in a patient-derived xenograft mouse model of small cell lung cancer. The concomitant use of EZH2 inhibitors has been found to prevent the emergence of acquired resistance, thus augmenting the chemotherapeutic efficacy^28^. Moreover, decreased phosphorylation of receptor tyrosine kinase by EZH2 inhibition results in the recovery of sunitinib anti-tumor effects in clear cell renal cell carcinoma^29^. Based on this knowledge, we analyzed the alterations in H3K27me3 level in HCC cells after exposures to sorafenib. Unexpectedly, sorafenib treatment induced an increase in H3K27me3 levels without remarkable changes in EZH1/2 expression.

Recently, there has been increasing evidence that the phosphorylation of various amino acid residue sites in EZH2 affect its methyltransferase activity^30^. These modifications occasionally cause the activation or suppression of enzymatic activity due to cell types and conditions. Among them, the phosphorylation of EZH2 at threonine 487 (Thr487) by CDK1/2, at Thr311 by AMPK, and at Ser21 by Akt, all inhibit the methyltransferase activity of EZH2^31–33^. Given that an increase in H3K27me3 levels caused by sorafenib treatment was accompanied by a decrease in the levels of phospho-Akt, we then examined the EZH2 phosphorylation status at Ser21. Immunocytochemical analyses and Western blotting analyses demonstrated a remarkable decrease in the number of phopho-EZH2 (Ser21)-expressing cells. Considering that decreased mRNA expression of histone demethylase UTX was observed in HCC cells treated with sorafenib, these results show that Akt suppression following sorafenib treatment increases H3K27me3 level through decreased EZH2 phosphorylation at Ser21. Co-treatment with sorafenib with either GSK126 or UNC1999 canceled the enhancement in H3K27me3 levels. These drugs synergistically inhibited cell proliferation in vitro and tumor growth in xenograft mouse models. Additionally, immunohistochemical analyses of xenograft tumors in which sorafenib was administrated for 6 weeks, showed similar results to those observed in the Western blotting of cultured cells. Collectively, the results suggest the possibility that the machinery was associated with sorafenib resistance in HCC. Because the hepatocyte-specific *EZH1/2* double knockout mice have been generated^34^, HCC mouse model using these mice might be a powerful tool to investigate the role of H3K27me3 in sorafenib treatment of HCC.

Irrespective of these findings, the Ser21 phosphorylation of EZH2 by Akt promotes carcinogenesis through methylation of the androgen receptor (AR) or AR-related proteins^35^. Phosphorylated EZH2 at Ser21 was reported to activate STAT via its methylation, and to enhance the tumorigenic potential of glioma stem-like cells^36^. The role of phosphorylated EZH2 in the methylation of non‐histone proteins would be further examined.

In conclusion, our study demonstrated that the combined use of sorafenib and EZH1/2 inhibitors exerted pronounced anti-tumor effects. Considering that the loss of PRC2 function was reported to cause RAS-MAPK signaling activation in peripheral nerve sheath tumors accompanied by NF1 mutations^37^, combined therapy with EZH1/2 inhibitors and MKIs might be reasonable. Given the many ongoing clinical trials of EZH2 and EZH1/2 inhibitors in a variety of cancers including HCC^38^, the combined use of epigenetic therapeutic agents, such as UNC1999, may prove useful to treat patients with advanced HCC.

## Materials and methods

### Reagents

UNC1999 was kindly provided by Prof. Jian Jin and Dr. Anqi Ma (Icahn School of Medicine at Mount Sinai). GSK126 and regorafenib were purchased from Cayman Chemicals (Ann Arbor, MI), whereas sorafenib, was purchased from LKT laboratories (Saint Paul, MN, USA).

### Cell culture and sphere formation assay

Human HCC cell lines (Huh7: CVCL_0336, HuH1: CVCL-2956, and PLC/PRF/5: CVCL_0485) and hepatoblastoma-derived cell line (HepG2: CVCL_0027) were obtained from the Health Science Research Resources Bank (Osaka, Japan). The cells were cultured in Dulbecco’s modified Eagle’s medium, containing 10% fetal bovine serum, 2 μM l-glutamine, 100 U/mL penicillin and 100 μg/mL streptomycin and all experiments were performed with mycoplasma-free cells. For the sphere formation assay, 1000 cells were plated onto ultra-low attachment 6**-**well plates (Corning, Corning, NY). The number of spheres (> 100 μm in diameter) was counted following 14 days of culture.

In the limiting dilution assays, cells ranging from 5 to 50 cells/well were seeded into ultra-low attachment 96 well plates (Thermo Fisher Scientific, Waltham, MA). After 7 days of culture, the percentage of wells without spheres (loge, Y-axis) for each cell plating density was calculated and plotted against the number of cells plated per well (X-axis). To calculate the sphere formation ability, the regression lines were plotted. These analyses were performed using the Extreme Limiting Dilution Analysis software (http://bioinf.wehi.edu.au/software/elda/).

### Trypan blue dye exclusion test

A total of 1 × 10^5^ HCC cells were plated onto 6-well plates. Cell growth was assessed by trypan blue staining after 48 and 96 h in culture. The combined effect of sorafenib with GSK126 or UNC1999 was analyzed by isobologram analysis using the Compu-Syn software program (ComboSyn, Inc., Paramus, NJ)^22^.

### Immunocytochemistry

Cells were fixated with 4% paraformaldehyde, after which they were blocked with normal goat serum, stained with anti-EZH2 (Cell Signaling Technology, Danvers, MA) and anti-H3K27me3 (Millipore, Billerica, MA) antibodies, and incubated with Alexa 488- or Alexa 555-conjugated immunoglobulin G (IgG) antibodies (Molecular Probes, Eugene, OR). To detect phosphorylated EZH2 after sorafenib treatment, the cells were stained with anti-phospho-EZH2 (Ser21, Abcam, Cambridge, MA), then incubated with Alexa-555-conjugated goat anti-rabbit IgG antibody (Molecular Probes, Eugene, OR). Apoptotic cells were evaluated by staining with anti-CASP3 antibody (Merck Millipore, Billerica, MA, USA), followed by Alexa-555-conjugated goat anti-rabbit IgG (Molecular Probes, Eugene, OR). Subsequently, the cells were coverslipped using a mounting medium that contained 4′,6-diamidino-2-phenylindole dihydrochloride (DAPI; Vector Laboratories, Burlingame, CA). Percentage apoptosis induction following 48 h treatment with GSK126 or UNC1999 was calculated by the fraction of CASP3 positive cells on 500 HCC cells.

### Viral production and transduction

Lentiviral vectors (CS-H1-shRNA-EF-1α-RFP) expressing short hairpin RNA (shRNA), which targets the human *EZH1* (target sequence: sh-*EZH1*-1, 5′-GGGATTTGCAAGCCAACATAT-3′; sh-*EZH1*-2, 5′-GGAGCTGATCAATAACTATGA-3′) were constructed. Lentiviral vectors (CS-H1-shRNA-EF-1α-EGFP) expressing shRNA, which targets the human *EZH2* and *luciferase (Luc)* was used for double knockdown of *EZH1* and *EZH2*^18^. Recombinant lentivirus vector (pLV[Exp]-EGFP:T2A:Puro) containing the coding sequence of human *EZH1* and *EZH2* were also produced (VectorBuilder Inc. Chicago, IL). The cells were transduced with viruses in the presence of protamine sulfate (10 μg/mL).

### Western blotting

Whole cell lysates were prepared using 0.1% NP-40 lysis buffer (20 mM sodium phosphate, pH 7.0, 300 mM NaCl, 5 mM EDTA and 0.1% NP40) supplemented with protease and phosphatase inhibitor cocktails (Roche) or sodium dodecyl sulfate (SDS)-sample buffer (25 mM Tris, pH 6.8, 1% SDS, 5% glycerol, 0.05% bromophenol blue and 1% β-mercaptoethanol). These lysates were separated by SDS-PAGE, transferred to a poly vinylidene di-fluoride (PVDF) membrane, and blotted by the following antibodies: anti-EZH1 (Abcam), anti-EZH2 (Cell Signaling Technology), anti-α-tubulin (Calbiochem, San Diego, CA), anti-H3 (Abcam), anti-H3K27me3 (Cell Signaling Technology), anti-ERK1/2 (Cell Signaling Technology), anti-phospho-ERK1/2 (Thr202/Tyr204, Cell Signaling Technology), anti-Akt (Cell Signaling Technology), anti-phospho-Akt (Ser473, Cell Signaling Technology), anti-PARP (Cell Signaling Technology), anti-phospho-EZH2 (Ser21, Abcam), anti-phospho-EZH2 (Thr311, Cell Signaling Technology). To detect tubulin and the protein of interest at the same time, some membranes are cut at around 75 kDa before hybridization with the antibody. The membranes were developed with Immobilon Western Chemiluminescent HRP substrate (EMD Millipore) and the signals were detected using ChemiDoc XRS systems (Bio-Rad Laboratories). The band intensity in Western blotting was totally quantified using Image Lab 6.0.1 software (Bio-Rad Laboratories). Relative intensity (RI) of target protein normalized to internal control was calculated. The entire image obtained is provided to Supplementary Figs. S5–S12.

### Quantitative real-time PCR

Total RNA was extracted using RNeasy plus Mini Kit or RNeasy plus Micro Kit (Qiagen). Subsequently, cDNA synthesis was performed using ThermoScript real-time polymerase chain reaction (RT-PCR) system (Invitrogen). Quantitative RT-PCR (RT-qPCR) was performed with a StepOnePlus RT-PCR System (Applied Biosystems) using the Universal Probe Library System (Roche Diagnostics) according to the manufacturer’s instructions. The relative expression was determined using the delta Ct method^39^. The primer sequences are listed in Supplementary Table S1.

### RNA sequencing

RNA sequencing was performed as previously described^40^. Briefly, total RNA purified from HCC cells was subjected to reverse transcription, and cDNA was synthesized using a SMARTer Ultra Low Input RNA Sequencing Kit (Clontech). cDNA libraries were generated using a NEBNext Ultra DNA Library Prep Kit (New England BioLabs), according to the manufacturer’s indications. After quantification of the libraries using Agilent 2100 Bioanalyzer (Agilent), sequencing was performed using a Hiseq1500 (Illumina). The RNA-sequence reads were aligned to the human reference genome (hg19 from University of California, Santa Cruz Genome Browser; http://genome.ucsc.edu/) using TopHat 1 (version 2.0.13; with default parameters). Gene expression values were calculated as reads per kilobase of exon unit per million mapped reads using Cufflinks (version 2.2.1).

### Xenograft transplantation

Six-week-old non-obese diabetic/severe combined immunodeficiency (NOD/SCID) male mice (Sankyo Laboratory Co. Ltd., Tsukuba, Japan) were bred and maintained according to our institutional guidelines for the use of laboratory animals. A total of 2 × 10^6^ Huh7 cells were implanted into the subcutaneous space of the mice’s backs. Sorafenib (5 mg/kg) was administrated daily by oral gavage, and UNC1999 (15 mg/kg) was administrated intraperitoneally 3 days a week for 6 weeks (n = 5 for each group). The treatment with sorafenib and/or UNC1999 was started the day after the cell transplantation. Subcutaneous tumors were subjected to hematoxylin and eosin (H&E) staining and immunohistochemistry with anti-EZH2, anti-phospho-EZH2 (Ser21), anti-phospho-Akt, anti-H3K27me3, anti-CASP3, and anti-Ki67 (DAKO, Carpinteria, CA) antibodies. TUNEL assay was also performed for the detection of apoptosis.

### Patients and surgical specimens

Surgical specimens were collected from 72 patients who underwent surgical resection for HCC at the Chiba University hospital between August 2009 and August 2015. A total of 72 pairs of tumor and tumor-free liver tissue were histologically examined. Paraffin embedded tumor sections and the surrounding tumor-free tissues were examined by H&E staining and immunohistochemistry with anti-EZH2 and anti-H3K27me3 antibodies. To investigate the mechanisms underlying the discrepancy between EZH2 expression and H3K27me3 levels, mRNA expression of *EZH1* and *UTX* in EZH2^low^H3K27me3^high^ tumors was analyzed by RT-qPCR.

### Statistical analysis

Data are presented as the mean ± standard error of the mean (SEM). The association between EZH2 expression and clinicopathological features was analyzed using Student's t-test for continuous variables (age, AFP levels, PIVKA-II levels, and tumor diameter) or the Chi-square test for categorical variables (gender, etiology, presence of liver cirrhosis, presence of portal involvement, Edmondson grade, and UICC stage). The relationship between EZH2 expression and H3K27me3 levels based on immunohistochemical staining were analyzed by Fisher’s exact test. Both growth of cells in cultures and tumor volume in xenograft models were analyzed by repeated measures analysis of variance (ANOVA). Recurrence-free survival (RFS) was calculated by the Kaplan–Meier method with the log–rank test. A *p-*value < 0.05 was considered statistically significant.

### Deposition of data

RNA sequence data were deposited in the DNA Data Bank of Japan (DDBJ) (accession number: DRA010957).

### Ethics statement

All animal procedures were performed according to Chiba University guidelines for the use of laboratory animals and approved by the review board for animal experiments of Chiba University. Our in vivo study was carried out in accordance with ARRIVE guidelines (https://arriveguidelines.org).

All patients provided informed consent and this study was approved by the Research Ethics Committees of the Graduate School of Medicine, Chiba University (approval number: 3024). We confirm that all procedures were performed in accordance with the relevant guidelines and regulations of the Declaration of Helsinki.

## Supplementary Information

Supplementary Information.
