# Supplementary material for: EZH1/2 inhibition augments the anti-tumor effects of sorafenib in hepatocellular carcinoma
Source: Sci Rep. 2021 Nov 1;11:21396. doi: 10.1038/s41598-021-00889-0 (PMC8560765; doi:10.1038/s41598-021-00889-0)
Supplement: Supplementary file 1 — Supplementary Information. [file 41598_2021_889_MOESM1_ESM.pdf]

EZH1/2 inhibition augments the anti-tumor effects of sorafenib in hepatocellular carcinoma

Authors:

Yuko Kusakabe [1], Tetsuhiro Chiba [1,*], Motohiko Oshima [2,3], Shuhei Koide [2,3], Ola Rizq [2,3], Kazumasa Aoyama [3], Junjie Ao [1], Tatsuya Kaneko [1], Hiroaki Kanzaki [1], Kengo Kanayama [1], Takahiro Maeda [1], Tomoko Saito [1], Ryo Nakagawa [1], Kazufumi Kobayashi [1], Soichiro Kiyono [1], Masato Nakamura [1], Sadahisa Ogasawara [1], Eiichiro Suzuki [1], Shingo Nakamoto [1], Shin Yasui [1], Rintaro Mikata [1], Ryosuke Muroyama [4], Tatsuo Kanda [5], Hitoshi Maruyama [6], Jun Kato [1], Naoya Mimura [7], Anqi Ma [8,9,10], Jian Jin [8,9,10], Yoh Zen [11], Masayuki Otsuka [12], Atsushi Kaneda [13], Atsushi Iwama [2,3], Naoya Kato [1]

Institutions:

[1] Department of Gastroenterology, Graduate School of Medicine, Chiba University, 1-8-1 Inohana, Chuo-ku, Chiba 260-8670, Japan

[2] Division of Stem Cell and Molecular Medicine, Center for Stem Cell Biology and Regenerative Medicine, The Institute of Medical Science, The University of Tokyo, 4-6-1 Shirokenadai, Minato-ku, Tokyo 108-8639, Japan

[3] Department of Cellular and Molecular Medicine, Graduate School of Medicine, Chiba University, 1-8-1 Inohana, Chuo-ku, Chiba 260-8670, Japan

[4] Department of Molecular Virology, Graduate School of Medicine, Chiba University, 1-8-1 Inohana, Chuo-ku, Chiba 260-8670, Japan

[5] Department of Gastroenterology and Hepatology, Nihon University School of Medicine, 30-1 Oyaguchi-Kamicho, Itabashi-ku, Tokyo 173-8610, Japan

[6] Department of Gastroenterology, Juntendo University School of Medicine, 2-1-1 Hongo, Bunkyo-ku, Tokyo 11308421, Japan

[7] Department of Transfusion Medicine and Cell Therapy, Chiba University, 1-8-1 Inohana, Chuo-ku, Chiba 260-8670, Japan

[8] Mount Sinai Center for Therapeutics Discovery, Icahn School of Medicine at Mount Sinai, New York, NY 10029, USA

[9] Department of Pharmacological Sciences, Icahn School of Medicine at Mount Sinai, New York, NY 10029, USA

[10] Department of Oncological Sciences, Tisch Cancer Institute, Icahn School of Medicine at Mount Sinai, New York, NY 10029, USA

[11] Institute of Liver Studies, King's College Hospital, London, United Kingdom

[12] Department of General Surgery, Graduate School of Medicine, Chiba University, 1-8-1 Inohana, Chuo-ku, Chiba 260-8670, Japan

[13] Department of Molecular Oncology, Graduate School of Medicine, Chiba University, 1-8-1 Inohana, Chuo-ku, Chiba 260-8670, Japan

Supplementary Table S1. Primer sequences designed for quantitative real-time PCR

Gene name	Sequence	
EZH1	Fw	5'- CATCCAGCGTGGACTTAAGAA -3'
	Rv	5'- CGTTCTTCTGCACAGACTCCT -3'
EZH2	Fw	5'- AGCTCCCGCTGAGGATGT -3'
	Rv	5'- CAGTGTGCAGCCCACAAC -3'
UTX	Fw	5'- CATGAACACAGCACAGCAGA -3'
	Rv	5'- ACCATGAATGAGCTTGTTGCT -3'
GAPDH	Fw	5'- CTGACTTCAACAGCGACACC -3'
	Rv	5'- TAGCCAAATTCGTTGTCATACC -3'

Fw, Forward, Rv, Reverse

Kusakabe et al. Supplementary Figure S1

A

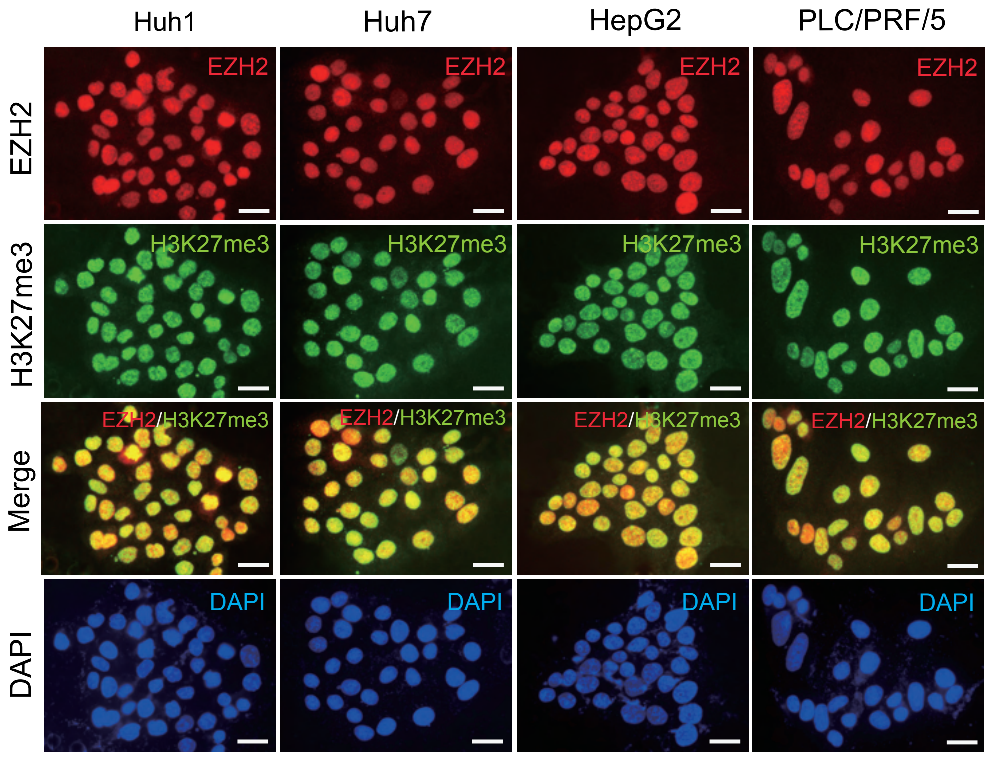

B

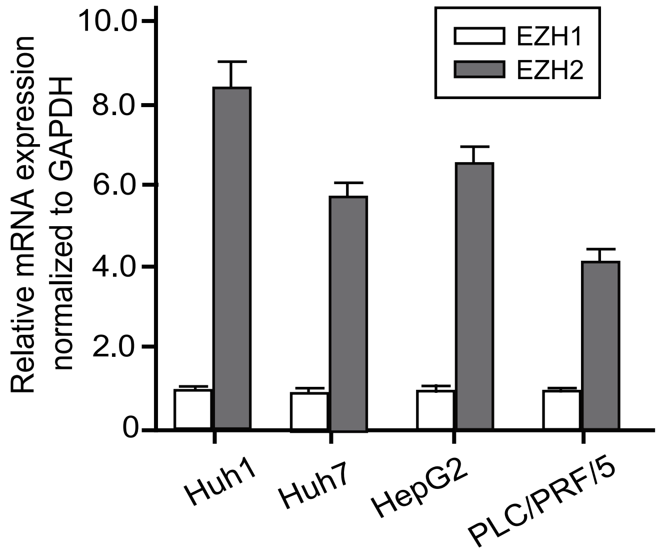

Supplementary Figure S1. Basal levels of EZH1, EZH2, and H3K27me3 in the HCC cell lines, Huh1, Huh7, HepG2, and PLC/PRF/5 cells. (A) Immunocytochemical analyses for EZH2 (red), H3K27me3 (green), and DAPI (blue). Scale bar = 20 µm. (B) RT-qPCR analyses of the expression of EZH1/2 in HCC cells.

Kusakabe et al. Supplementary Figure S2

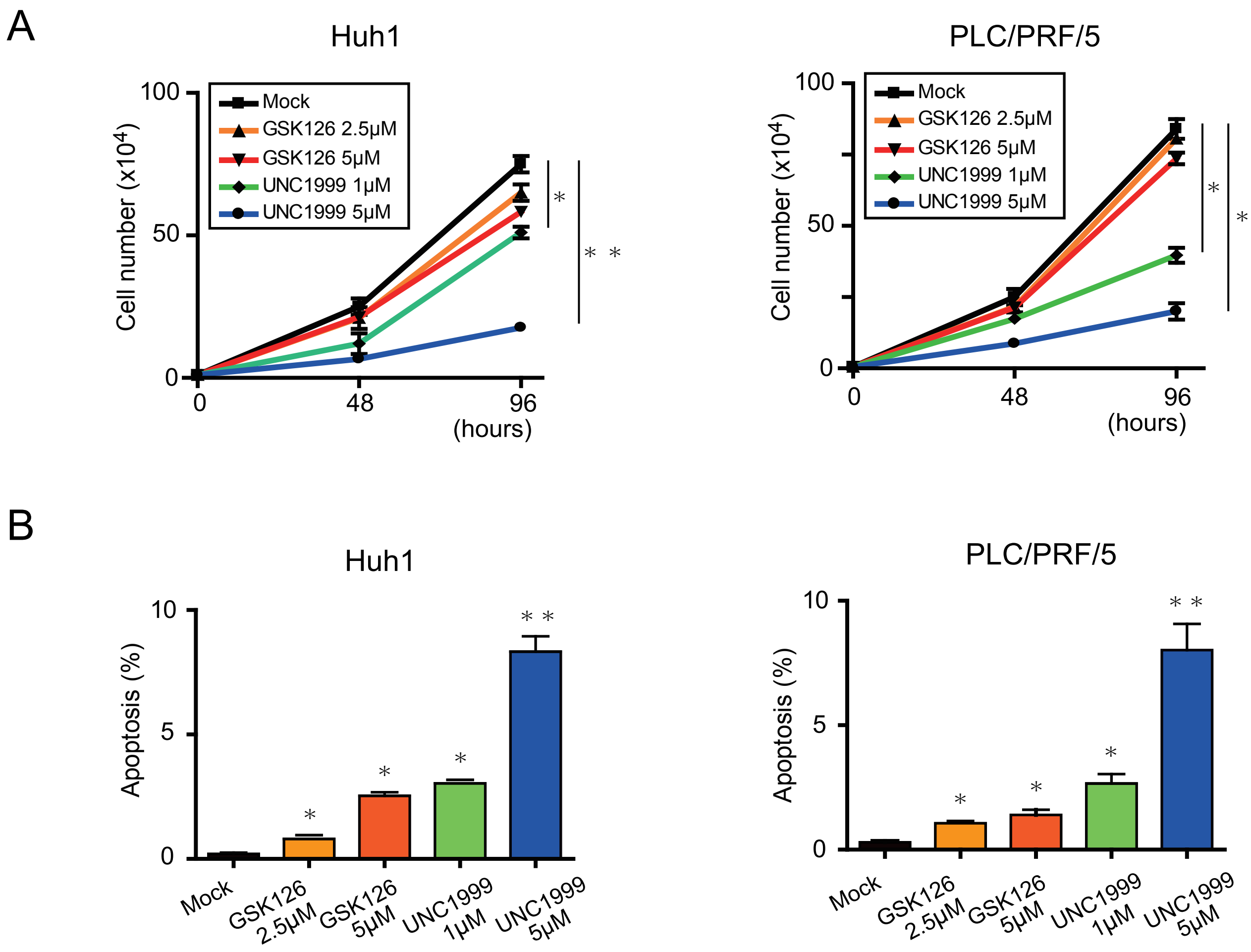

Supplementary Figure S2. In vitro assays of Huh1 and PLC/PRF/5 cells treated with GSK126 and UNC1999. (A) Inhibition of cell growth in HCC cells treated with GSK126 and UNC1999 in a dose- and time-dependent manner (repeated measures ANOVA, **p* < 0.05, ***p* < 0.01).

(B) Apoptotic induction after treatment of GSK126 and UNC1999 based on CASP3-positive cells.

Kusakabe et al. Supplementary Figure S3

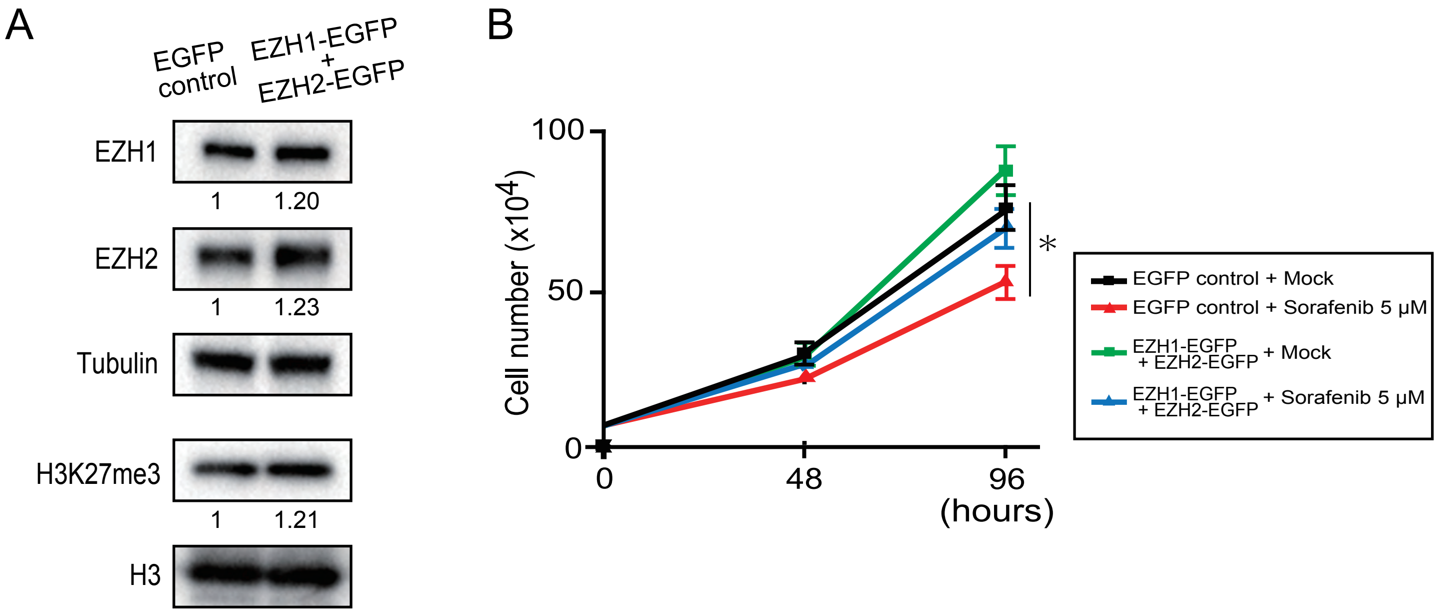

Supplementary Figure S3. Sorafenib treatment in EZH1/2-overexpressed HCC cells.
(A) EZH1/2-overexpressed cells were subjected to Western blot analyses. RI was shown as the means of three independent experiments. (B) Cell growth inhibition by sorafenib in EZH1/2-overexpressed cells (repeated measures ANOVA, *$p < 0.05$).

Kusakabe et al. Supplementary Figure S4

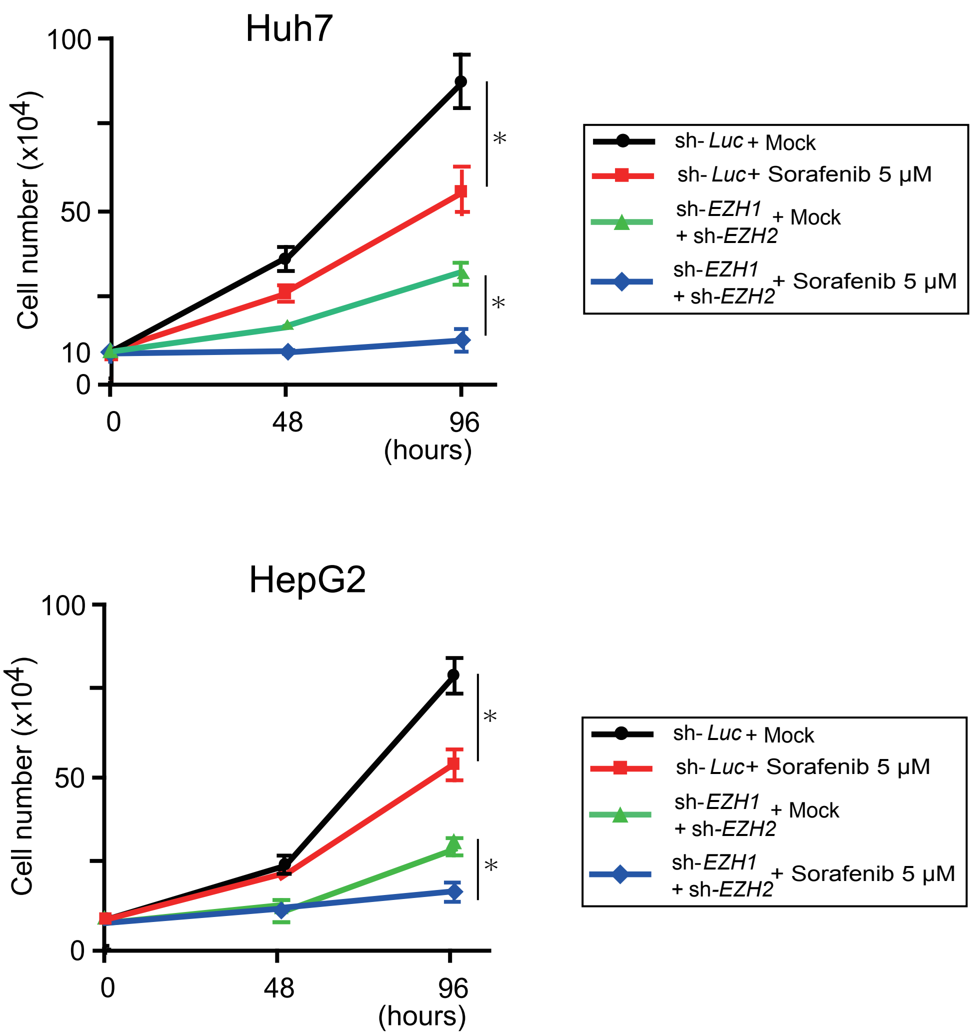

Supplementary Figure S4. Sorafenib treatment in EZH1/2-knockdown cells. Cell growth inhibition by sorafenib in EZH1/2-knockdown cells (repeated measures ANOVA, *$p < 0.05$)

Kusakabe et al. Supplementary Figure S5

Fig. 2A

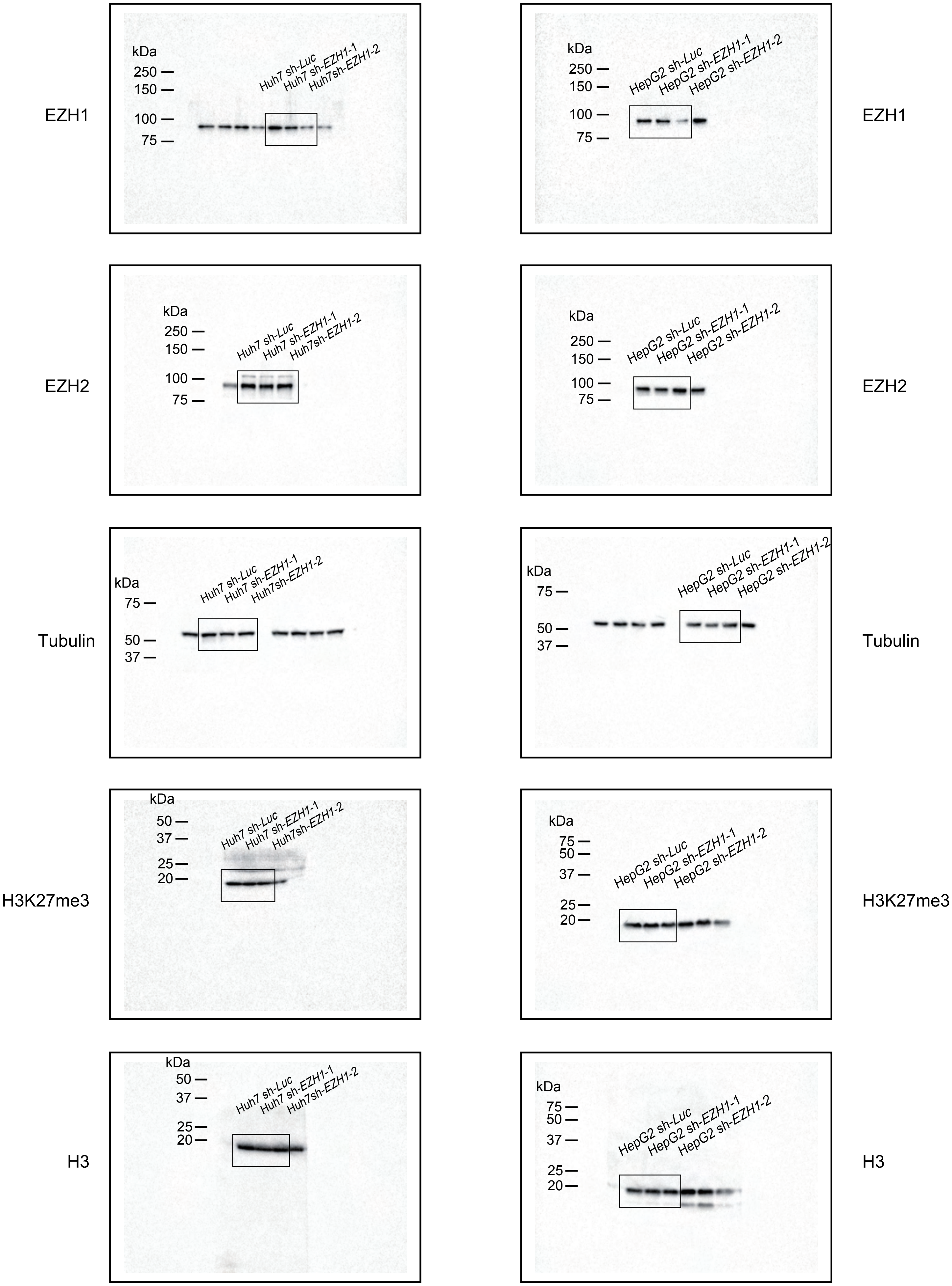

Supplementary Figure S5. Uncropped Western blot images of Figure 2A.

Kusakabe et al. Supplementary Figure S6

Fig. 2B

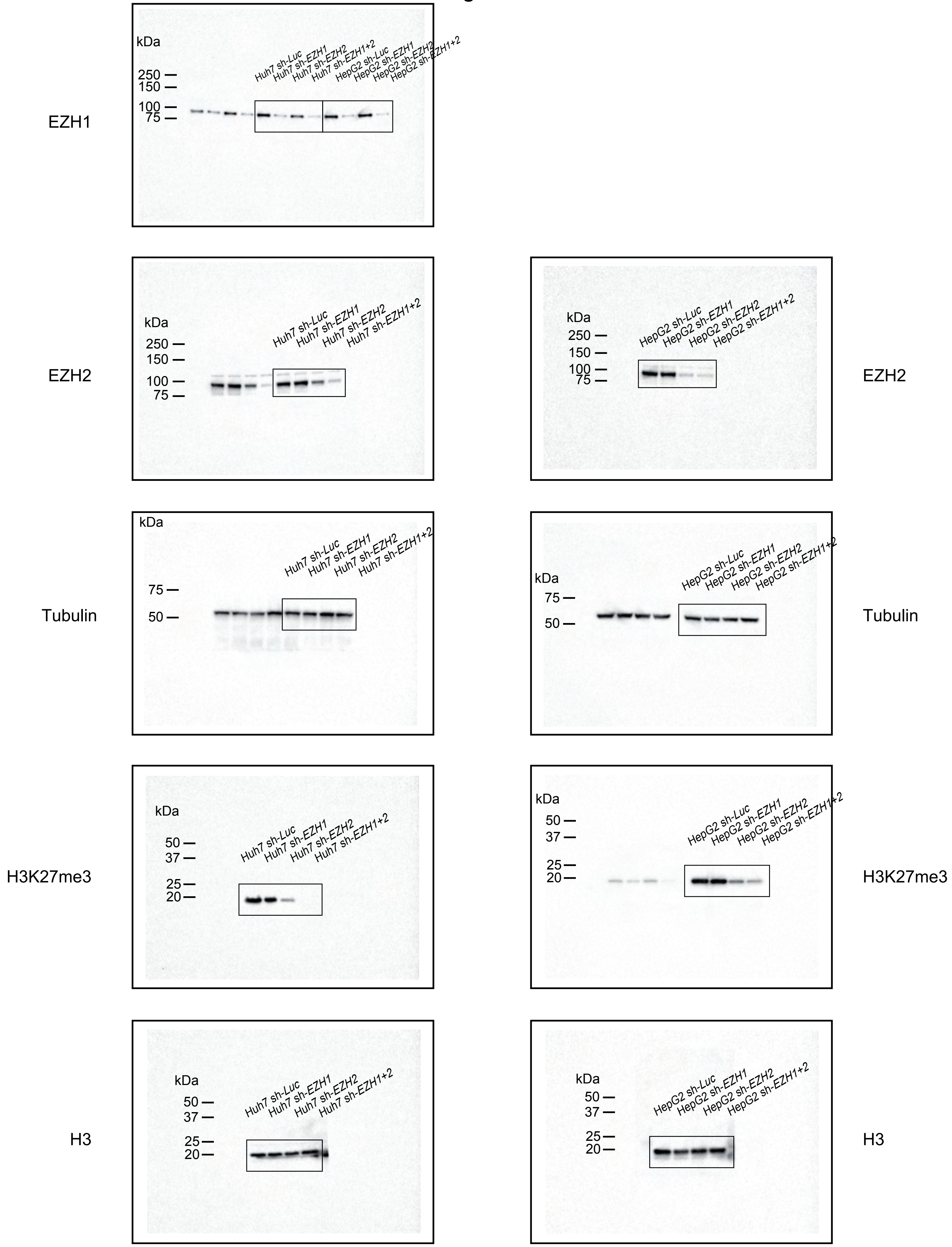

Supplementary Figure S6. Uncropped Western blot images of Figure 2B.

Kusakabe et al. Supplementary Figure S7

Fig. 3D : Huh7

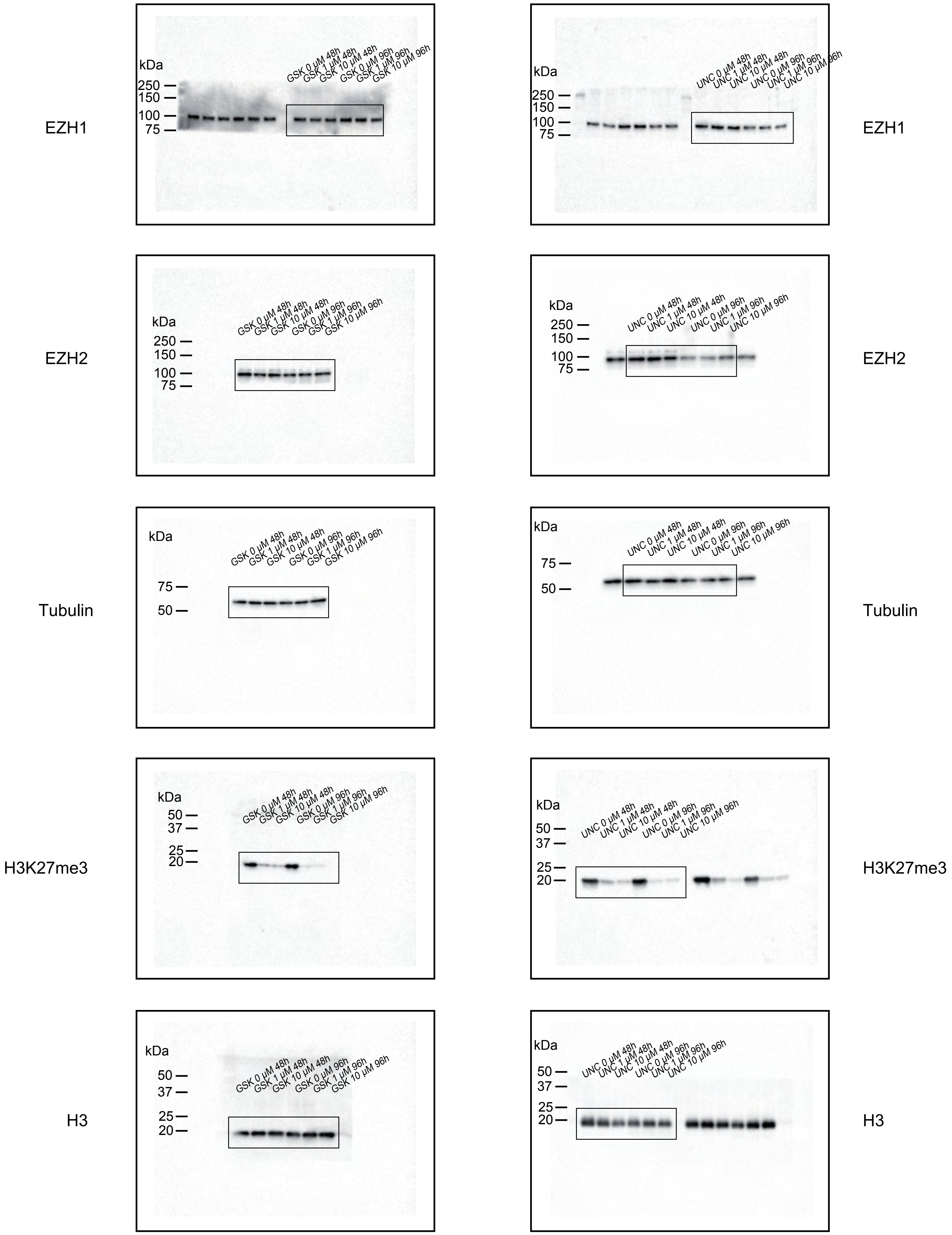

Supplementary Figure S7. Uncropped Western blot images of Figure 3D (Huh7).

Kusakabe et al. Supplementary Figure S8

Fig. 3D : HepG2

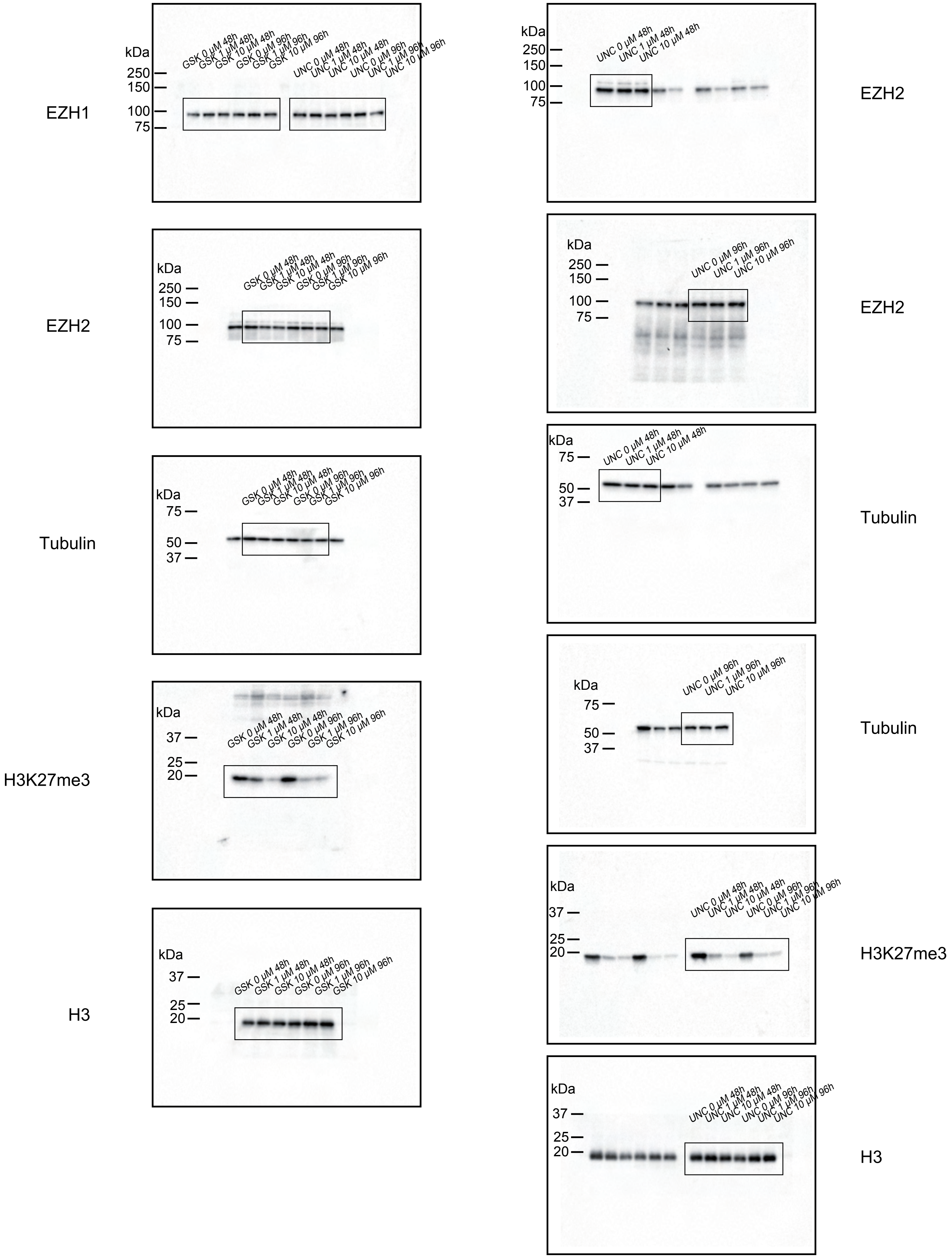

Supplementary Figure S8. Uncropped Western blot images of Figure 3D (HepG2).

Kusakabe et al. Supplementary Figure S9

Fig. 4A : Huh7

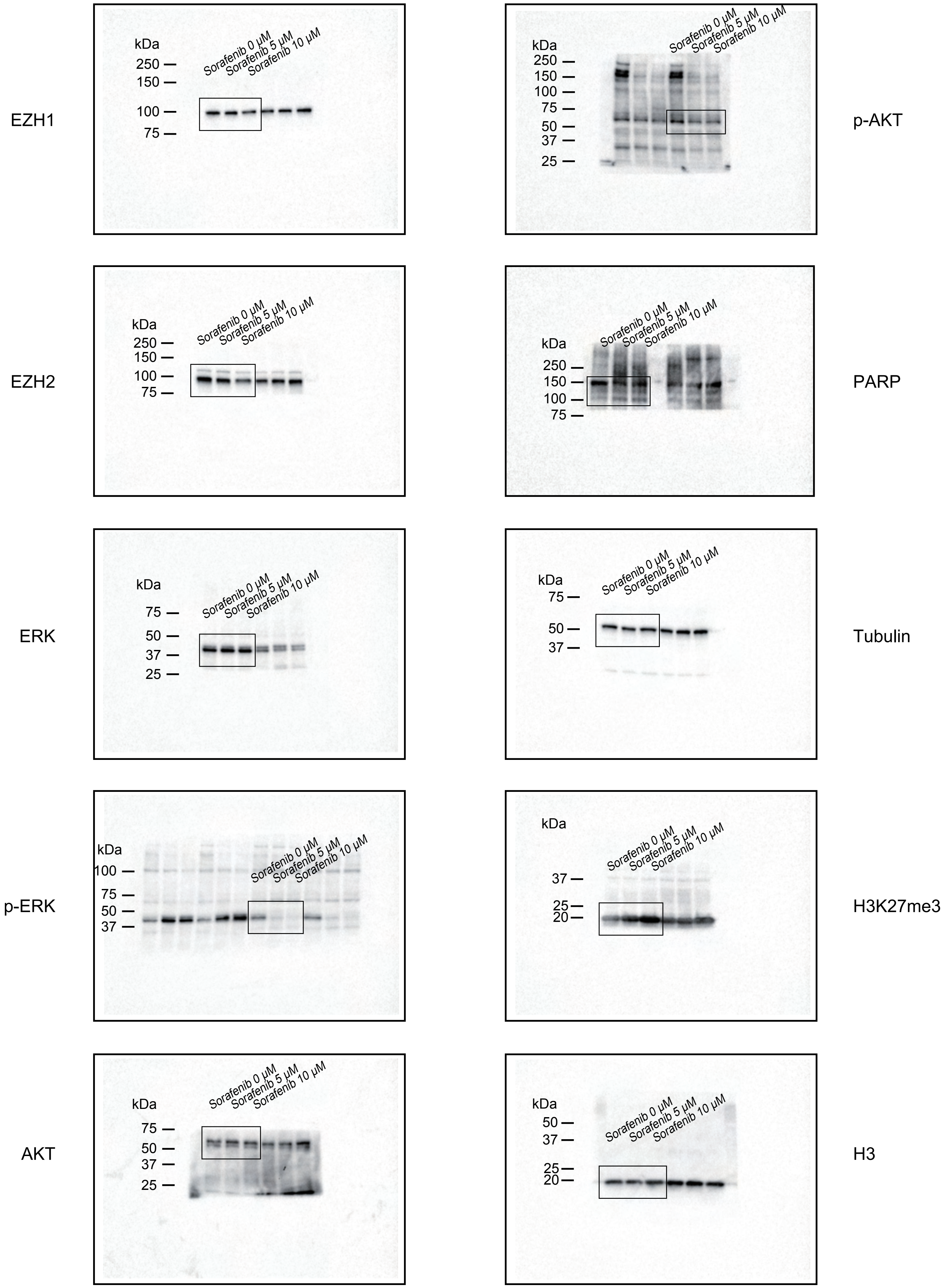

Supplementary Figure S9. Uncropped Western blot images of Figure 4A (Huh7).

Kusakabe et al. Supplementary Figure S10

Fig. 4A : HepG2

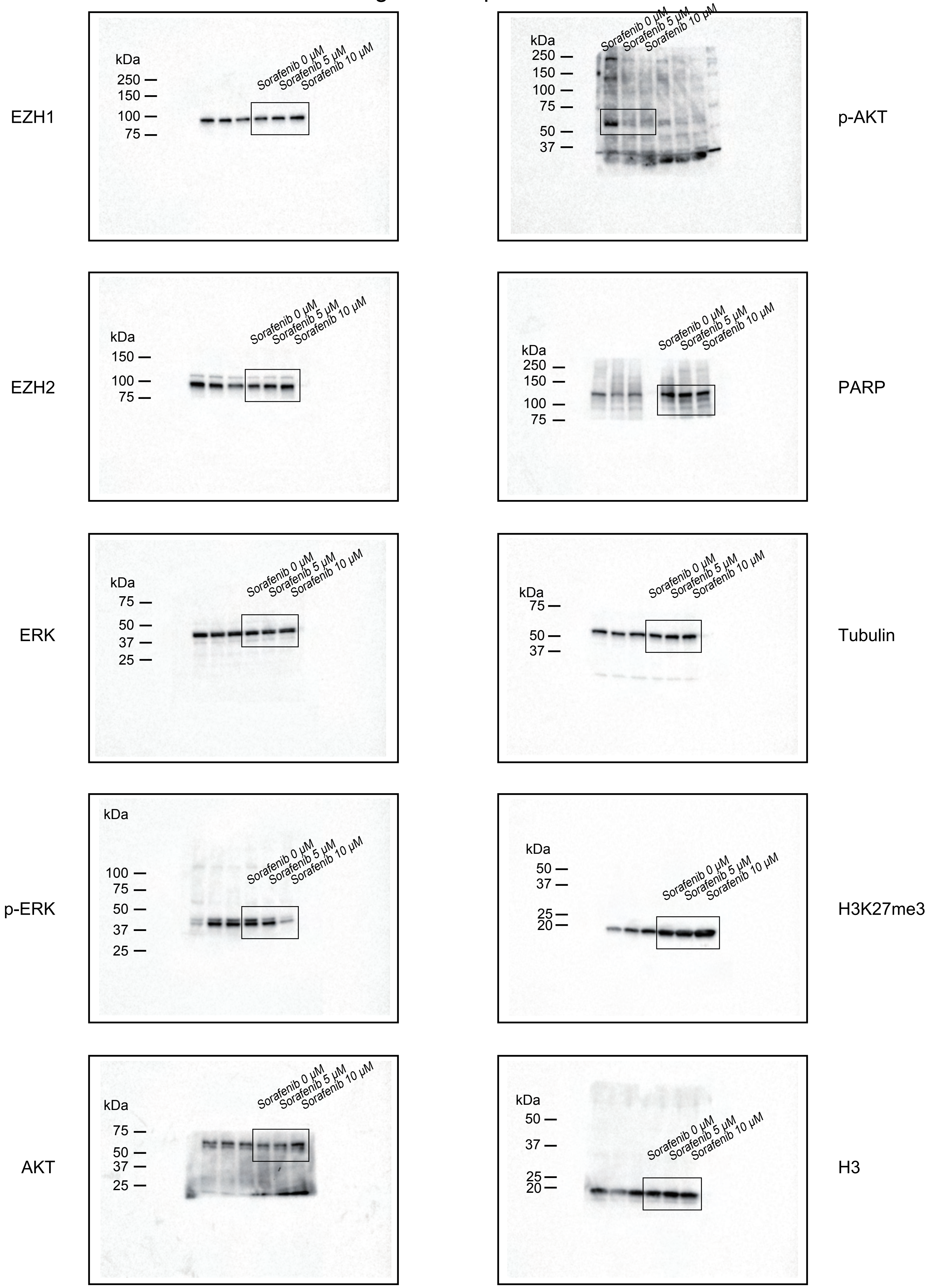

Supplementary Figure S10. Uncropped Western blot images of Figure 4A (HepG2).

Kusakabe et al. Supplementary Figure S11

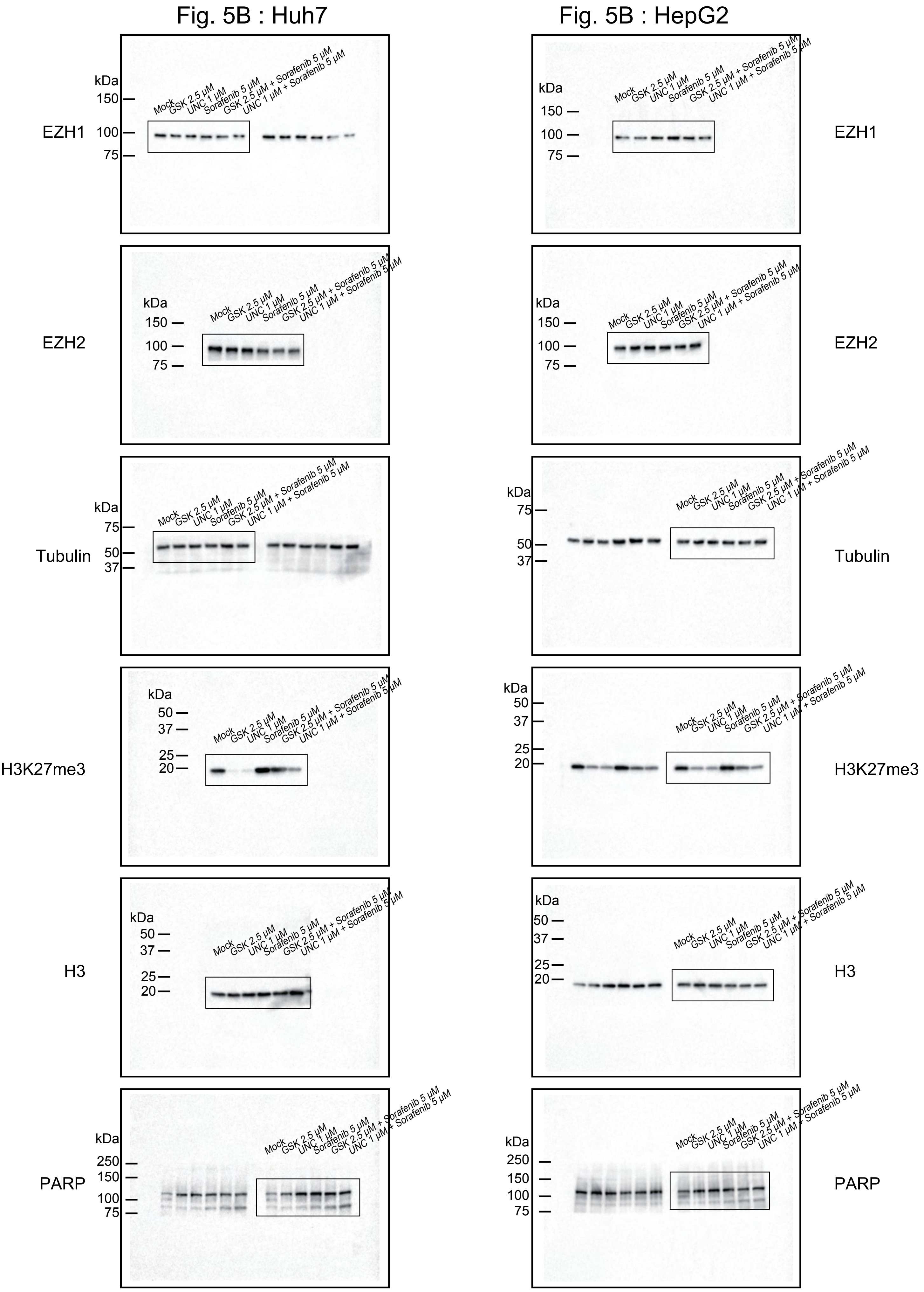

Supplementary Figure S11. Uncropped Western blot images of Figure 5B.

Kusakabe et al. Supplementary Figure S12

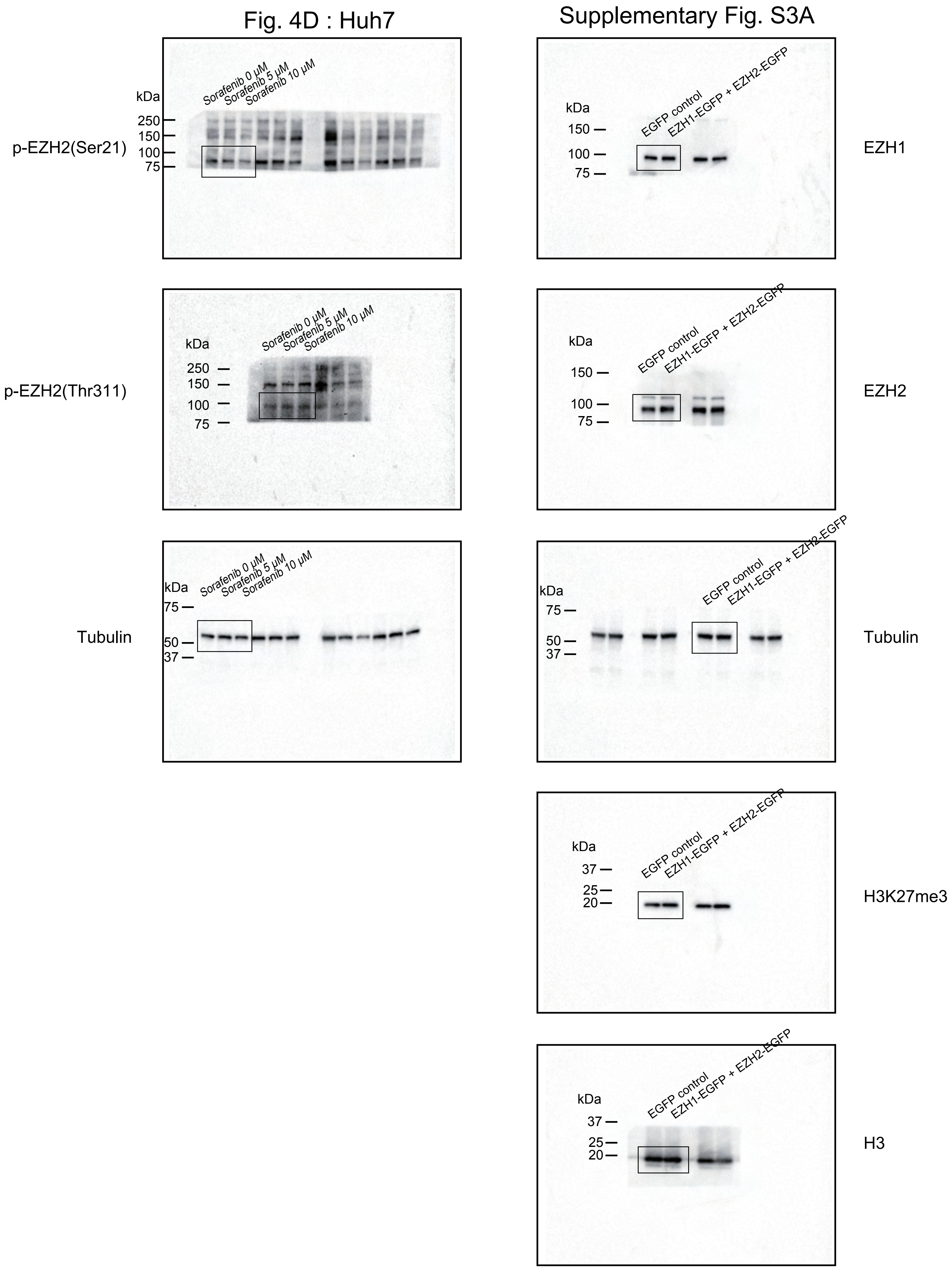

Supplementary Figure S12. Uncropped Western blot images of Figure 4D and Supplementary Figure S3A.
